# Synergistic Effects of High-Intensity Ultrasound Combined with L-Lysine for the Treatment of Porcine Myofibrillar Protein Regarding Solubility and Flavour Adsorption Capacity

**DOI:** 10.3390/foods13040629

**Published:** 2024-02-19

**Authors:** Yongkang Xie, De Chen, Jianxin Cao, Xuejiao Wang, Xiaoyu Yin

**Affiliations:** College of Food Science and Engineering, Kunming University of Science and Technology, Kunming 650500, China; xieyongkang1234@126.com (Y.X.); cd17852035931@163.com (D.C.); jxcao321@hotmail.com (J.C.); wangxuejiao173@hotmail.com (X.W.)

**Keywords:** ultrasound, low ion strength, amino acid, myofibrillar proteins, flavour, conformation

## Abstract

This study aimed to investigate the synergistic effects of high-intensity ultrasound (0, 5, 10, 15, and 20 min) in combination with L-lysine (15 mM) on improving the solubility and flavour adsorption capacity of myofibrillar proteins (MPs) in low-ion-strength media. The results revealed that the ultrasound treatment for 20 min or the addition of L-lysine (15 mM) significantly improved protein solubility (*p* < 0.05), with L-lysine (15 mM) showing a more pronounced effect (*p* < 0.05). The combination of ultrasound treatment and L-lysine further increased solubility, and the MPs treated with ultrasound at 20 min exhibited the best dispersion stability in water, which corresponded to the lowest turbidity, highest absolute zeta potential value, and thermal stability (*p* < 0.05). Based on the reactive and total sulfhydryl contents, Fourier transform infrared spectroscopy, and fluorescence spectroscopy analysis, the ultrasound treatment combined with L-lysine (15 mM) promoted the unfolding and depolymerization of MPs, resulting in a larger exposure of SH groups on the surface, aromatic amino acids in a polar environment, and a transition of protein conformation from α-helix to β-turn. Moreover, the combined treatment also increased the hydrophobic bonding sites, hydrogen-bonding sites, and electrostatic effects, thereby enhancing the adsorption capacity of MPs to bind kenone compounds. The findings from this study provide a theoretical basis for the production and flavour improvement of low-salt MP beverages and the utilisation of meat protein.

## 1. Introduction

Meat and meat products are nutrient-rich and essential components of the daily hu-man diet [1]. Meat proteins contain essential amino acids required by the human body, which are easily digested and absorbed by the body [2]. Generally, meat is ingested in its entirety as a solid mass, necessitating adequate mastication and digestive capabilities. Nevertheless, meat and meat products are inherently limited in their appeal to older individuals, dysphagic patients, and individuals with gastrointestinal and digestive disorders, which is primarily due to their impaired chewing and digestive systems, which restricts their ability to consume normal crude fibre meat [3]. Hence, there is a need to offer weakened protein in a fluid form to ensure adequate nutrient intake and satiety among this demographic [4].

Myofibrillar proteins (MPs) are the most important structural proteins in muscle, accounting for 50–60% of meat proteins. Myosin and actin are the main proteins in MPs [5]. MPs are more soluble in high-salt environments and are primarily responsible for the water holding and emulsifying ability of the meat [4]. Low-ion-strength media will significantly reduce the solubility and dispersibility of MPs and prevent the unfolding of their structures, thereby limiting their application in low-salt fluid diets [6,7]. However, excessive salt intake can bring about a series of chronic diseases, which seriously endanger human health. Therefore, it can be considered that improving the solubility and dispersion stability of MPs in low-ion-strength solutions is a prerequisite for the development of MP beverages and the expansion of meat protein utilisation [8]. Currently, many chemical (amino acid, acid or alkaline solubilization, etc.) or physical methods (ultra-high pressure, ultrasound, etc.) are available to assist in the processing of MPs to improve their solubility and dispersion stability in low-ion-strength solutions [4,9]. Moreover, researchers have paid increasing attention to the combination of two or more assistive methods (ultrasound-assisted glycation, high pressure combined with CaCl_2_, etc.) to ameliorate the solubility of MPs [4]. Amino acids are naturally occurring compounds in meat [1]. The addition of amino acids to food is beneficial to maintain or improve the solubility of MPs [10]. Guo et al. [11] found that the introduction of arginine and L-lysine induced changes in the secondary as well as tertiary structures of the protein. The hydrophobic and sulfhydryl groups buried inside the MPs were exposed to the surface, thus increasing the solubility of the MPs. The addition of proline under low-salt conditions also resulted in an increase in MP solubility [10]. However, according to the current reports, the highest solubility can reach approximately 40% by adding amino acids, and there is still a definite gap from complete dissolution [12,13]. Moreover, the flavour of protein solutions with added amino acids is also a problem [4].

High-intensity ultrasound (HIU) is a non-ionising, non-invasive, non-polluting, and green non-thermal form of mechanical energy that has been widely applied in protein processing [14]. In recent years, many studies have demonstrated the ability of ultrasound to improve the solubility of MPs [4]. The cyclic growth and violent collapse of cavitation bubbles during ultrasonication cause high temperature, pressure, high-shear energy waves, and turbulence in the cavitation zone, which alters the structural properties of the treated protein molecules [14]. Liu et al. [8] reported that HIU with different power levels could improve the solubility as well as the dispersion stability of MPs. It was also hypothesised that physical disruption of the myosin filamentous structure may be the main reason for the increase in MP solubilization. Moreover, ultrasound has good potential for improving the flavour quality of meat products [15]. MPs can bind to flavour compounds through chemical forces such as hydrogen bonds, van der Waals forces, and hydrophobic interactions [16]. The cavitation effects of ultrasonic dissociates water molecules produces highly active hydroxyl radicals and further changes and modifies the structure and intermolecular forces of MPs, thereby affecting the flavour adsorption capacity of MPs [17]. It has been reported that different ultrasound powers can enhance the binding of MPs to furan flavour compounds [18,19]. However, the investigation of amino acids combined with ultrasound treatment for the enhancement of the solubility of MPs in low-ion-strength media has not been reported yet. Moreover, little is known about the effect on the binding ability of MPs to flavour compounds after both treatments, which are still worth exploring.

In this study, the aim was to investigate the potential of high-intensity ultrasound combined with L-lysine to improve the solubility and flavour adsorption capacity of MPs in low-ion-strength media. The synergistic effects of different ultrasound times combined with L-lysine on the pH, solubility, turbidity, particle size, and zeta potential of MPs in low-ion-strength media were investigated. The structure characterization of MPs and the binding capacity between MPs and four ketone compounds were analysed. Finally, the thermal stability of MPs treated with ultrasound and L-lysine was evaluated. This study is expected to provide a theoretical basis for the production and flavour improvement of low-salt MP beverages and the expansion of meat protein utilisation.

## 2. Materials and Methods

### 2.1. Materials and Reagents

Fresh longissimus muscle was purchased from a local Yonghui supermarket (Kunming, Yunnan Province, China), which was obtained from pig carcasses of approximately 6 months of age at 24 h post-mortem following standard industrial procedures. 2-Pentanone, 2-hexanone, 2-heptanone, and 2-octanone were purchased from Dr. Ehrenstorfer (Dr. Ehrenstorfer, Schwerin, Germany). The purity of the standard flavour compounds was between 95.50% and 99.60%. L-lysine, potassium sodium tartrate, sodium chloride, and sodium hydroxide were purchased from Aladdin (Shanghai, China). All chemical reagents used in this study were of analytical grade or higher.

### 2.2. Preparation of MPs

MPs were extracted from the longissimus muscle after the removal of fat and connective tissue using the method of Chen et al., with modifications [20]. Briefly, approximately 200 g of minced meat was thoroughly homogenized with 4 vol. (*w*/*v*) of 10 mM phosphate buffer (0.1 M NaCl, 2 mM MgCl_2_, 1 mM EGTA, pH 7.0) for 60 s at 4 °C, and then the mixture was centrifuged at 2000× *g* for 15 min. The pellet was collected and washed twice with 4 vol. (*w*/*v*) of the same phosphate buffer and centrifugation conditions as above. After centrifugation, the MP pellet was washed three times with 4 vol. (*w*/*v*) of salt solution (0.1 M NaCl) under the same centrifugation conditions as mentioned above. The suspension underwent filtration using four layers of cheesecloth during the final wash, and then the pH was modified to 6.0 using a 0.1 M HCl solution. The final MP concentration was calculated using the Biuret method with bovine serum albumin as a standard [21]. The MPs were kept at 4 °C for use within 48 h.

### 2.3. Sample Treatment

In total, 50 mL of MP solution (5 mg/mL, diluted with 0.01 M phosphate buffer (0.1 M NaCl, pH 7.0) was placed in a double-walled beaker (100 mL) for every ultrasonication time [19]. An ultrasonic processor (SCIENTZ-IID, Ningbo Xinzhi Bio-technology Co., Ltd., Ningbo, China) equipped with a titanium probe with a diameter of 6.0 mm was inserted in the equivalent amounts of MP solutions with and without adding L-lysine at a depth of 1.5 cm from the bottom. The concentration of L-lysine solution for this study was 15 mM. Ultrasound treatment was conducted at a frequency of 20 kHz with different ultrasonic times (0, 5, 10, 15, and 20 min) at 300 W (pulse duration of 2 s on and 3 s off), and all the samples were treated at low temperatures (4–10 °C).

### 2.4. Solubility Measurements

The solubility of MP solution (20 mL) with and without adding L-lysine treated at different ultrasound times was measured as described by Shen et al. [22]. MP solution (5 mg/mL) was centrifuged at 5000× *g* for 20 min, the supernatant was collected, and the protein content was determined with the Biuret method. Protein solubility was expressed as the percentage of protein concentration in the supernatant after centrifugation relative to the initial protein concentration.

### 2.5. Turbidity Measurements

The turbidity of the MP solution (20 mL) with and without adding L-lysine treated at different ultrasound times was analysed using the method of Wang et al. [23], with modifications. The MP solution (1 mg/mL) was measured with a UV spectrophotometer (UV-1800PC, Mepda Shanghai, China) at 350 nm and 25 °C. The turbidity of MPs was expressed as their absorbance.

### 2.6. pH Measurements

The pH value of the MP solution (20 mL) with and without adding L-lysine treated at different ultrasound times was measured with a pH metre (FE28, Mettler Toledo, Zurich, Switzerland).

### 2.7. Particle Size and Zeta Potential Measurements

The particle size of the MP solution (10 mL) with and without adding L-lysine treated at different ultrasound times was measured with the Malvern MasterSizer 3000 (Malvern Instruments Ltd., Worcestershire, UK). The zeta potential was measured using the method of Chen et al. [24], with some slight modifications. The MP solution was diluted to a final concentration of 0.1 mg/mL using 0.01 M phosphate buffer (0.1 M NaCl, pH 7.0), injected into the Marvin Zeta potentiometer (Zetasizer Nano ZS90, Malvern, UK), and measured at 25 °C.

### 2.8. Fourier Transform Infrared Spectroscopy (FT-IR)

Infrared spectroscopy was carried out using the method of Kaewprachu et al. [25], with a slight modification. The samples were vacuum freeze-dried and then placed in a Fourier transform infrared spectrometer (TENSOR27, Bruker, Ettlingen, Germany) after KBr compression (1:50) with a scanning range of 4000–400 cm^−1^ and 64 scans with a resolution of 4 cm^−1^, with KBr as the background single channel. The secondary structural contents of the protein samples were calculated using Peakfit 4.12 software.

### 2.9. Measurements of Reactive and Total Sulfhydryl Contents

The contents of reactive and total sulfhydryl groups were measured via the method of Ellman et al. [26], with some modifications. Briefly, 1 mL of sample (2 mg/mL) was thoroughly mixed with 8 mL of Tris-glycine buffer solution (86 mmol Tris, 92 mM glycine, 4 mM EDTA, 8 M urea, pH 8.0). The insoluble proteins were then removed via centrifugation at 10,000× *g* for 15 min. The supernatant was taken as 4.5 mL and reacted with 0.5 mL of Ellman’s reagent (4 mg/mL DTNB). The absorbance at 412 nm was measured after 30 min using a UV spectrophotometer. The same method was used to measure reactive group content, but urea was omitted from the glycine buffer solution.

### 2.10. Fluorescence Measurements

The endogenous fluorescence spectra were determined with reference to the method of Guo et al. [27], with a slight modification. The MP solution (5 mL) was diluted to 0.5 mg/mL and placed in a fluorescence spectrophotometer (F-7000, Hitachi Corp., Tokyo, Japan) to determine the endogenous fluorescence spectra with an excitation wavelength of 280 nm and an emission wavelength of 300–400 nm. The slit width was 10, and the smoothing degree was 20.

### 2.11. Differential Scanning Calorimetry (DSC)

The thermal stability of the sample was measured with differential scanning calorimetry (DSC) (Mettler Toledo, Zurich, Switzerland). A nitrogen flow rate of 20 mL/min was set. MP samples (5–10 mg) in aluminium DSC trays were heated from 30 °C to 120 °C at a heating rate of 10 °C/min. The blank control was an empty aluminium box to enable us to obtain the DSC curve.

### 2.12. Solid-Phase Microextraction (SPME) and Gas Chromatography–Mass Spectrometry (GC/MS) Analysis

The four selected ketones (2-pentanone, 2-hexanone, 2-heptanone, and 2-octanone) were dissolved in methanol to form a stock solution of each ketone. Then, each ketone was added to the treated MP dispersion solution. The concentration of each ketone in the MP solution was set to 10 mg/g. The control was carried out by replacing the MP solution with an equal volume of phosphate buffer. The protein–ketone mixture was filled into 20 mL headspace vials and sealed with PTFE-faced silicone septa (Supelco, Bellefonte, PA, USA). The protein and control samples were equilibrated at room temperature (25 °C) for 16 h. Subsequently, the amount of headspace ketone in each vial was measured with SPME-GC/MS (QP2010, Shimadzu, Japan) according to the method reported by Zhou et al. [28], with modifications. The flavour compounds were extracted with an SPME fibre at 30 °C for 30 min. Subsequently, the flavour compounds absorbed were dissolved in the GC inlet at 240 °C for 5 min. The initial GC oven temperature was held at 40 °C for 3 min, increased at 3 °C to 90 °C, held for 5 min, and then increased at 25 °C to 230 °C, and finally held at 230 °C for 5 min. Results were determined by the difference in peak area of the flavour compounds between the protein and control vials and were calculated using the following equation:

Free flavour compound (%) = (peak area protein/peak area control) × 100%

### 2.13. Statistical Analysis

Three independent batches of MPs (replicates) were prepared, and the experimental measurements were conducted in triplicate (triplicate observations) for each batch of MPs. The results were expressed as mean ± standard deviation (SD). The data were analysed using the General Linear Model procedure in the Statistix 8.1 software package (Analytical Software, St. Paul, MN, USA). The solubility, turbidity, pH, particle size, zeta potential, reactive and total sulfhydryl contents, and flavour adsorption capacity were analysed using two-way analysis of variance (ANOVA) in the factorial design procedure, which considered the ultrasound time, L-lysine (added or not), and their interactions as fixed effects and each replicate as a random effect. The determination of significant differences (*p* < 0.05) among the means was performed using Duncan’s multiple comparisons.

## 3. Results and Discussion

### 3.1. Protein Solubility

**Figure 1:**
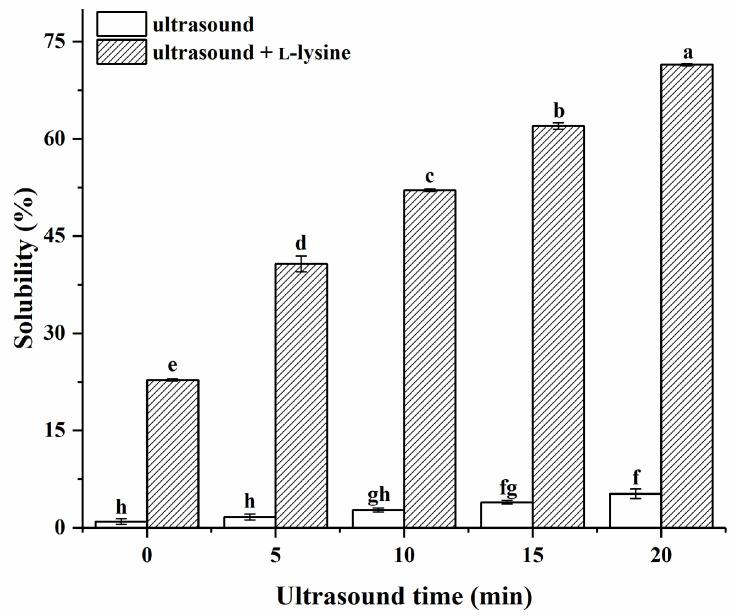
Effects of ultrasound combined with L-lysine on the solubility of myofibrillar proteins. The letters (a–h) above the bars indicate a significant difference (*p* < 0.05).

As presented in Figure 1 and Table S1, ultrasound time, L-lysine (added or not), and their interactions exerted significant effects on protein solubility (*p* < 0.05). As anticipated, the untreated MPs (control) exhibited comparatively limited solubility in low-ion-strength media, at only 0.96%. The obtained results exhibited conformity with prior studies, emphasising the lack of solubility of naive MPs in low-ion-strength media [8]. The solubility of MPs only treated with ultrasound gradually increased with the increase in ultrasound time (*p* > 0.05) and reached a maximum value of 5.25% at 20 min, which was significantly higher than that of the untreated MPs (*p* < 0.05). This may be since ultrasound promotes the unfolding of myosin filaments [29]. The solubility of MPs with L-lysine alone without ultrasonic treatment (0 min) was significantly increased (22.81%). Myosin is an important component of MPs [5]. The effects of L-lysine on myosin solubility were previously reported, where a low ionic strength of L-lysine at 0.1–0.2 M had a significant effect on solubility, while a high ionic strength between 0.25 and 0.3 M had a negligible effect on solubility [12]. The presence of L-lysine was reported to inhibit myosin aggregation, possibly by interacting with acidic amino acid residues of the myosin, thereby leading to an increase in myosin solubility [30]. The solubility increased significantly (*p* < 0.05) after ultrasound combined with L-lysine treatment of MPs versus MPs with L-lysine alone without ultrasonic treatment (0 min). It was worth noting that under the same ultrasound treatment time, the solubility of MPs treated with L-lysine combined with ultrasound in low-ion-strength media was superior to that of the MPs treated with L-lysine alone without ultrasonic treatment (0 min) or with ultrasound treatment alone. The ultrasound could promote the dissolution of myosin filaments and allow them to better unfold through cavitation, such as shear force, turbulence effect, and high-velocity fluid microjet [31], as well as facilitate the interactions of protein molecules and L-lysine, leading to better solubility. This was also evidenced by the interaction of the data. As the ultrasound time increased from 0 to 20 min, the solubility of MPs with the addition of L-lysine in low-ion-strength media showed a progressive increase to a maximum of 71.42%. According to this result, the effects of MP solubility enhancement depended on ultrasound time.

### 3.2. Protein Turbidity

**Figure 2:**
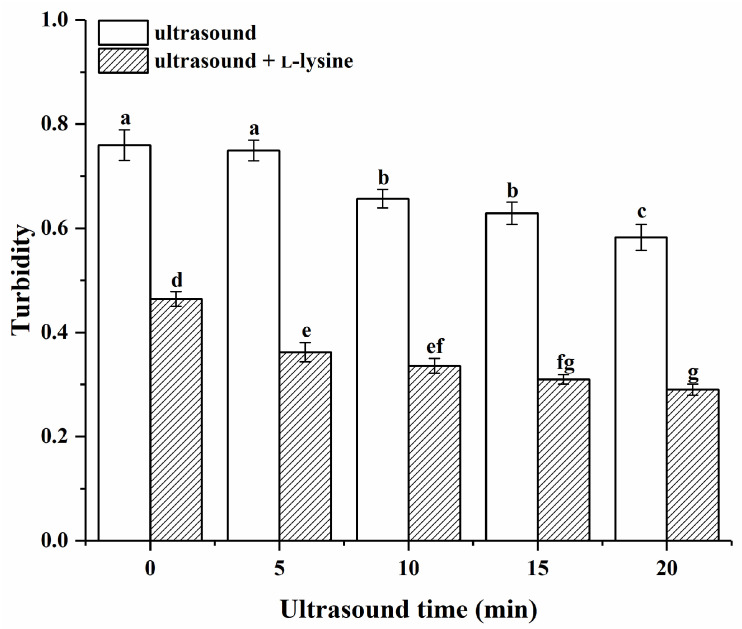
Effects of ultrasound combined with L-lysine on the turbidity of myofibrillar proteins. The letters (a–g) above the bars indicate a significant difference (*p* < 0.05).

As presented in Figure 2 and Table S1, ultrasound time, L-lysine (added or not), and their interactions exerted significant effects on the protein turbidity (*p* < 0.05). The turbidity of the untreated MPs (control) was high at 0.76. The turbidity of MPs only treated with ultrasound gradually decreased with the increase in ultrasound time (*p* < 0.05), which indicated that ultrasound could make the MP solution more clear and transparent, and the MP dispersion more uniform and consistent. The MP turbidity of L-lysine alone without ultrasonic treatment (0 min) was significantly lower than the turbidity of MP treated with ultrasound only (*p* < 0.05). Previous studies [32] have reported that the addition of L-lysine also led to a reduction in turbidity. After combining L-lysine with ultrasound treatment, the solubility of MPs was further decreased, stabilised, and reached a minimum value of 0.26 at 20 min. The turbidity of MPs treated with L-lysine and ultrasound in low-ion-strength media was lower than the turbidity of MPs treated with L-lysine alone without ultrasonic treatment (0 min) and the turbidity of MPs treated with ultrasound only at the same ultrasound treatment time. This may be due to a series of physical forces caused by the cavitation effects of ultrasound on the solution system, which breaks up protein molecules into smaller pieces. Also, ultrasonic binding of L-lysine may inhibit protein–protein covalent and noncovalent crosslinking and lower turbidity [31].

### 3.3. pH of Protein

The pH value is an important factor influencing the solubility of proteins [33]. The effects of ultrasound combined with L-lysine on the pH of MPs is presented in Table 1. The pH of MPs only treated with ultrasound did not change significantly with the increase in ultrasound time (*p* > 0.05), suggesting that ultrasound did not affect the pH and that pH was not the main factor contributing to the improvement of the solubility of MPs. This was consistent with the findings of Liu et al. [8], who hypothesised that HIU may provide a strong physical force through cavitation, disrupting the highly ordered filamentous myocardin structure and reducing the particle sizes of MPs, leading to an increase in the solubility of MPs. L-lysine is an alkaline amino acid [34], and the pH values of MPs were increased significantly by adding L-lysine (*p* < 0.05). As the ultrasound time increased, the pH values of MPs with the addition of L-lysine in low-ion-strength media showed no significant changes (*p* > 0.05). According to Yongsawatdigul et al. [35], myosin solubility was found to vary in a “U” shape with the pH value, with the minimum solubility generally corresponding to a pH between 4.0 and 6.0. Near the isoelectric point (pH = 5.4), myosin rapidly aggregated and precipitated, resulting in smaller solubility due to the reduction of intermolecular electrostatic repulsion. However, the solubility of MPs was higher near extreme acid/base conditions. The change in pH is an important factor in the solubility of MPs.

### 3.4. Particle Size of Protein

The particle size of protein varies with the degree of protein aggregation [29]. As shown in Table 2, the mean particle size D_43_ of the untreated MPs (control) was relatively large, with a wide particle distribution. Consistent with the results of Li et al. [36], certain chicken breast MPs including well-organised filamentous myosin persisted in the liquid suspensions. The particle size D_43_ of MPs only treated with ultrasound gradually decreased with the increase in ultrasound time (*p* < 0.05). This may be because under the operation of the ultrasound probe, cavitation and shear forces are accompanied by microfluidics and turbulence, which disrupt the integrity of myofibrils and the dissociation of proteins [37]. The particle size D_43_ of MPs of L-lysine alone without ultrasonic treatment (0 min) was larger than that of the untreated MPs (*p* < 0.05), which showed that there were swollen myofibrils. The phenomenon of myofibril swelling in salt solutions is well-established, since it is caused by the selective binding of CL-anions to myofibrils, resulting in an increase in electrostatic repulsion between them [30]. However, one possible explanation for how L-lysine causes myofibrils to swell is that when myofibrils swell, the overall density of myofibrils decreases, which may make MPs less likely to form a precipitate during gentle centrifugation [31]. After combining L-lysine with ultrasound treatment, the D_43_ of MPs decreased. However, it is higher than that of the ultrasound treatment group alone (*p* < 0.05), which may be because the effects of L-lysine on particle size was greater than that of ultrasound on particle size. After the introduction of ultrasonic treatment, the D_32_ and D_43_ of MPs decreased by 50.26% and 59.16% as the ultrasonic time increased from 0 min to 20 min, respectively, suggesting that ultrasound contributed to the depolymerization of MPs. This is a result of the physical force that ultrasound can produce in breaking up protein particles, which reduces the size of the protein [36].

### 3.5. Zeta potential of Protein

**Figure 3:**
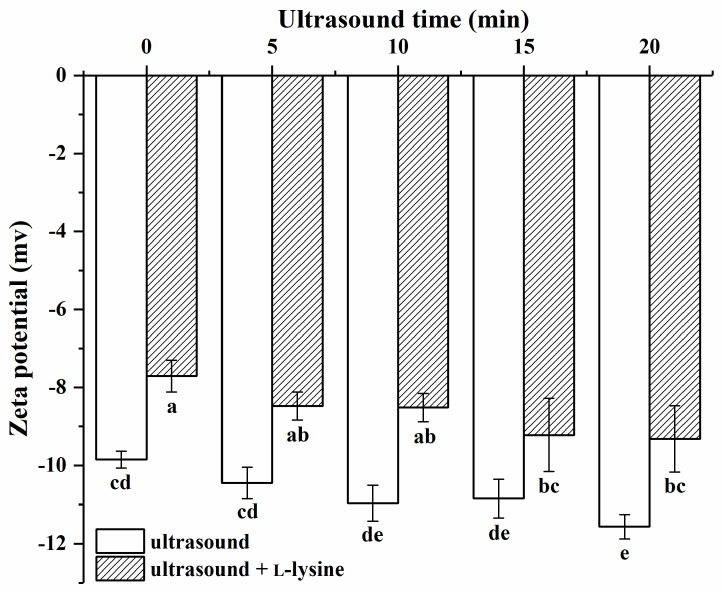
Effects of ultrasound combined with L-lysine on the zeta potential of myofibrillar proteins. The letters (a–e) above the bars indicate a significant difference (*p* < 0.05).

The zeta potential can reflect the charge that the MP surface is carrying and is crucial for maintaining the system’s stability [38]. Ultrasound combined with L-lysine affected the zeta potential of MPs in Figure 3. The zeta potential of all samples was negative, indicating a greater number of positive amino acids was present on the protein surface [39]. The absolute value of the potential of MPs treated with ultrasound gradually increased with the increase in ultrasound time (*p* > 0.05), and reached its absolute maximum value at 20 min, which was significantly higher than that of the untreated protein (*p* < 0.05). The increase in the absolute value of the potential of MPs after ultrasound treatment may be due to the destruction of protein aggregates via ultrasound treatment, resulting in an increase in specific surface area and an increase in surface negative charge [40]. Relevant studies have shown that the change in protein potential may have a relationship with protein particle size [38]. Compared to the untreated MPs, the particle size of the MPs’ fraction of L-lysine alone without ultrasonic treatment (0 min) increased, thereby decreasing the absolute value of the MPs’ potential. After combining L-lysine with ultrasound treatment, the absolute value of the potential of MPs further increased (*p* < 0.05). It was still lower than that of the MPs treated with ultrasound treatment alone (*p* < 0.05). Protein particles can disintegrate due to the physical force of ultrasound, which reduces their size and increases their potential [41]. However, in this study, the influence of L-lysine on particle size exceeded that of ultrasound.

### 3.6. Fourier Transform Infrared Spectroscopy (FT-IR) Analysis

FT-IR spectra provide valuable information regarding the absorption energy of chemical bonds, making them effective for the structural and chemical characterization of proteins [42]. The amide I band (1600–1700 cm^−1^) has become the most sensitive for predicting the secondary structural components of proteins in the infrared spectral region [43]. According to Table 3, the α-helix and β-turn contents of MPs treated only with ultrasound decreased slowly as the ultrasound time increased (*p* > 0.05), reaching their lowest points of 22.18% and 26.86% at 20 min, respectively. However, the β-sheet content gradually decreased (*p* < 0.05). The reason for this may be that the cavitation effect and microjet effect produced by ultrasound promoted the unfolding of protein molecules and weakened the hydrogen bonds, resulting in the disruption of the ordered structure of α-helix and β-turn, and the formation of β-sheet and a random coil. The ultrasound treatment induced conformational transitions from ordered to disordered structures [42]. The α-helix, random coil, and β-sheet of MPs were decreased by the addition of L-lysine only (*p* < 0.05). The decrease in the α-helix content indicated a change in protein conformation, thus gradually exposing the hydrophobic sites buried inside the molecule [44]. The MPs treated with L-lysine and ultrasound had no significant effect on the content of secondary structures (*p* > 0.05), which may be due to the greater effects of L-lysine on the secondary structures than ultrasound. As a result, the effects of ultrasound on the secondary structures became less significant. Guo et al. [11] found that α-helix was significantly lost after the introduction of L-lysine, regardless of ionic strength. It has also been hypothesised that the mechanism for the increase in myosin solubility is the ability of the L-lysine cation to bind to the negatively charged residues of myosin via electrostatic interaction, disrupting intra- and intermolecular ionic linkages [11]. This results in a conformational shift of the protein, loss of α-helical structure, and exposure of hydrophobic and masked SH groups to the surface. Myosin filaments depolymerize, ultimately increasing protein solubility [11].

### 3.7. Total and Reactive Sulfhydryl Analysis

**Figure 4:**
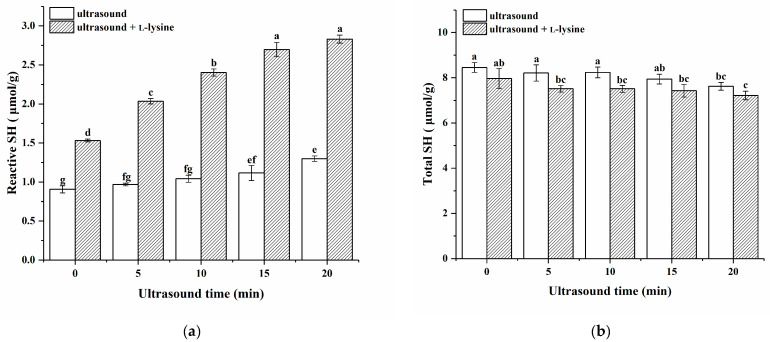
Effects of ultrasound combined with L-lysine on the contents of reactive sulfhydryl groups (**a**) and total sulfhydryl groups (**b**) in myofibrillar proteins. The letters (a−g) above the bars indicate a significant difference (*p* < 0.05).

The sulfhydryl group is an important reactive group that reflects structural alterations in MPs [45]. The effects of ultrasound combined with L-lysine on the contents of total and reactive sulfhydryl contents in MPs are shown in Figure 4. The total sulfhydryl content of MPs treated with ultrasound alone gradually decreased with increasing ultrasound time (*p* > 0.05) and reached a minimum value of 7.62 μmol/g at 20 min, which was significantly higher than that of the untreated MPs (*p* < 0.05). These results indicated that some free sulfhydryl groups may be converted to disulfide bonds, hence enhancing the crosslinking between protein subunits [19]. The total sulfhydryl content of the MP of L-lysine alone without ultrasonic treatment (0 min) did not change significantly (*p* > 0.05). Guo et al. [11] reported similar results in the content of total sulfhydryl groups when investigating the effects of L-lysine and L-histidine on the solubility and conformational properties of porcine myosin. After combining L-lysine with ultrasound treatment, the total sulfhydryl content of MPs treated with ultrasound for 20 min was significantly lower than that of untreated MPs (*p* < 0.05). Ultrasound treatment decreased the total sulfhydryl content, probably because the physical forces generated via ultrasound disrupt the structure of the protein molecules, exposing the sulfhydryl groups originally buried inside to the surface, and the enhancement of hydrophobic interactions reduces the intermolecular sulfhydryl distances [46]. When the protein solution is exposed to oxygen, the more reactive sulfhydryl groups and shorter distances lead to the formation of disulfide bonds, which results in a decrease in total sulfhydryl content [47].

The reactive sulfhydryl content of the MPs treated with ultrasound alone exhibited a gradual increase with the extension in ultrasound duration (*p* < 0.05), which is similar to the study by Zhao et al. [19]. This observation can be attributed to the ultrasound treatment-induced boost in reactive sulfhydryl content, which facilitated the unfolding of protein structures and exposed internal functional groups such as sulfhydryl groups. An increase in reactive sulfhydryl content was observed when L-lysine was used alone without ultrasonic treatment (0 min), indicating that SH groups were exposed to the protein surface. After combining L-lysine with ultrasound treatment, the reactive sulfhydryl content of MPs further increased. It is worth noting that under the same ultrasound treatment time, the reactive sulfhydryl content of MPs treated with L-lysine combined with ultrasound in low-ion-strength media is significantly higher than that of MPs treated with L-lysine alone without ultrasonic treatment (0 min) and with ultrasound alone. This finding suggests that ultrasound treatment in combination with L-lysine further amplifies the exposure of SH groups on the protein surface. This phenomenon can be attributed to the synergistic effects of ultrasound and L-lysine, which promotes the unfolding of protein molecules and stimulates the production of hydroxyl radicals. Furthermore, this indicated that the addition of L-lysine does not disrupt the formation or cleavage of disulfide bonds but rather reveals the concealed SH groups on the protein surface [19].

The sulfhydryl group is a common site within protein molecules that is sensitive to oxidation [48]. Under conditions of oxidative stress, the sulfhydryl group is highly susceptible to attack by oxidising agents, leading to the formation of disulfide bonds [48]. The oxidative modifications of sulfhydryl groups are considered significant indicators of protein oxidation [49]. Specifically, a decrease in sulfhydryl group content often indicates an increased level of protein oxidation [50]. In this study, after combining L-lysine with ultrasound treatment, the total sulfhydryl content of ultrasound-treated MPs for 20 min was significantly lower than that of untreated MPs (*p* < 0.05), suggesting that the combination of these two factors promotes protein oxidation. While previous research has mainly focused on the negative effects of protein oxidation on the quality of meat products, recent studies have indicated that protein oxidation can also have positive influences on the quality characteristics of meat and related products to some extent. Moderate oxidative conditions can have a beneficial impact on the processing and quality characteristics of meat products. Liu et al. [51] discovered that under appropriate oxidation conditions, protein oxidation enhances protein sensitivity to enzymes, facilitating their digestion and absorption in the human body. Zhou et al. [28] used a hydroxyl radical oxidation system to perform oxidative modifications on MPs and observed that targeted oxidant concentrations significantly enhanced the binding capacity of MPs to specific flavour compounds. This enhancement contributes to the preservation of flavour in meat products throughout the processing stages. Furthermore, there have been reports indicating that HIU promotes the oxidation of proteins and lipids in meat, which is consistent with the results of our study. According to Antti et al. [52], the cavitation effects of ultrasound results in the decomposition of water molecules, leading to the generation of highly reactive hydroxyl radicals. These radicals further facilitate the formation of strong oxidising H_2_O_2_, causing significant oxidation of lipids and proteins in meat. Consequently, the oxidation reactions of proteins and lipids contribute to the formation of flavour compounds such as aldehydes and ketones [53]. Moreover, it has been documented that varying ultrasound powers can enhance the interaction between meat proteins and furan flavour compounds [19]. As a result, ultrasound-assisted processing techniques hold significant potential for improving the flavour quality of meat products.

### 3.8. Fluorescence Intensity Analysis

**Figure 5:**
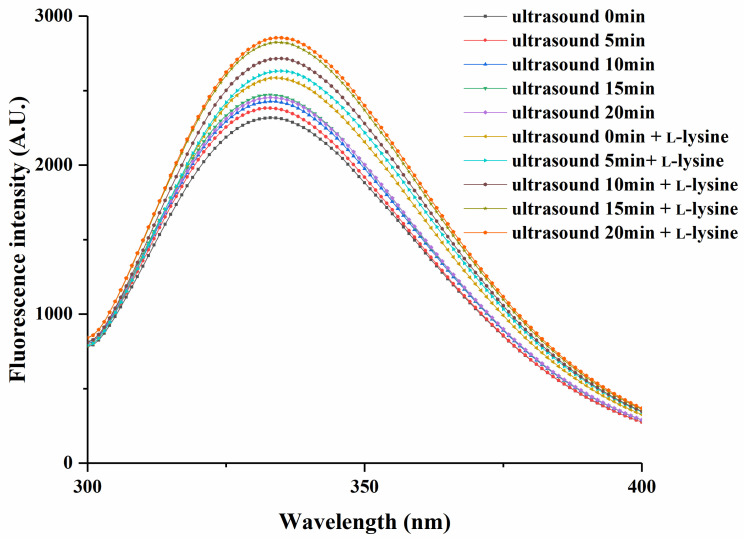
Effects of ultrasound combined with L-lysine on the tertiary structures of myofibrillar proteins.

Fluorescence spectra have been extensively utilised for the investigation of protein conformation [54]. The intrinsic fluorescence spectra of the protein microenvironment, wherein residues such as tryptophan (Trp) act as natural chromophores, enable us to characterize the changes in residue polarity [55]. Such changes in polarity can provide insights into the alterations in the tertiary structures of proteins [55]. As depicted in Figure 5, the maximum emission wavelength (λ max) of MPs treated only with ultrasound exhibited an increase from 332.4 nm to 333 nm as the ultrasound time increased from 0 to 20 min, concomitant with a rise in fluorescence intensity. The red shift of the peak indicated the unfolding of the tertiary structure of protein, leading to a greater exposure of aromatic amino acids to the polar environment [36]. Furthermore, the increased maximum fluorescence intensity suggested that the cavitation and mechanical effects induced by ultrasound enhanced the exposure of hydrophobic groups [56]. With L-lysine alone and without ultrasonic treatment (0 min), the λ max of MPs consistently exhibited a red shift, accompanied by a continuous increase in fluorescence intensity. This observation suggested that under the current conditions, L-lysine induced a further unfolding of protein [1]. Subsequently, when L-lysine was combined with ultrasound treatment, the λ max of MPs continued to increase with increasing ultrasound time. At 20 min, the maximum value of 334.8 nm was reached. This phenomenon can be attributed to the unfolding of MP molecules due to the combined application of ultrasound and L-lysine, resulting in the exposure of chromophores such as Trp to the solvent and subsequent solvent quenching, thus influencing the fluorescence intensity. Notably, both the fluorescence intensity and the λ max of the MPs treated with the combined treatment of L-lysine and ultrasound exceeded those of the MPs treated with L-lysine alone without ultrasonic treatment (0 min) or ultrasound alone at the same ultrasonication time in low-ion-strength media. These findings indicated the maximum level of unfolding in the tertiary structures of the protein molecules [3].

### 3.9. DSC of Protein

Differential scanning quantities are employed to analyse energy and conformational changes during protein denaturation [57]. The higher the protein denaturation temperature, the higher the thermal stability [57]. In Table 4, upon the addition of L-lysine alone without ultrasonic treatment (0 min), the heat denaturation temperature of the MPs was elevated to 109.5 °C. The rise in thermal stability can be attributed to the basic nature of L-lysine, which increased the pH value of the solution and consequently enhanced the heat denaturation temperature [58]. Furthermore, the combination of L-lysine with ultrasound resulted in an increased stability of MPs, reaching a maximum value of 114.5 °C at 20 min, which is consistent with Bi et al., who found that ultrasound treatment enhanced the thermal stability of chickpea protein [59]. Ultrasonication disrupted the tertiary structures of MPs through mechanical and shear forces, resulting in increased exposure of SH groups to the protein surface [8]. The total sulfhydryl group content also indicated that some free sulfhydryl groups may be converted to disulfide bonds, hence enhancing the crosslinking between protein subunits [19]. Additionally, the increased thermal stability of MPs may be due to forces that reduce the interactions after HIU treatment, such as internal hydrophobic groups. This led to the unfolding of buried internal hydrophobic groups and increased intermolecular interactions, resulting in the formation of thermally stable polymers [60]. HIU assisted in preventing protein re-agglomeration during post-sonication processing by producing smaller and more stable protein particles [59]. Furthermore, this significant improvement in thermal stability may originate from the interaction between the ultrasound, L-lysine, and protein. Therefore, the combined treatment of ultrasound and L-lysine demonstrates an ability to enhance the thermal stability of MPs [4].

### 3.10. Binding of MPs to Ketone Compounds

**Figure 6:**
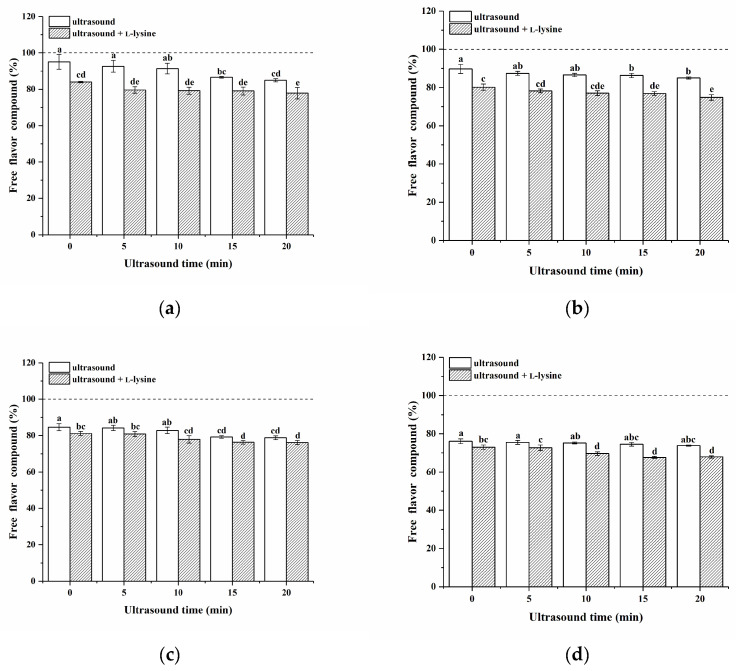
Effects of ultrasound combined with L-lysine treatment of pork myofibrillar proteins on the adsorption capacity of four ketones (2-pentanone, 2-hexanone, 2-heptanone, and 2-octanone). 2-pentanone (**a**), 2-hexanone (**b**), 2-heptanone (**c**), and 2-octanone (**d**). The letters (a–e) above the bars indicate a significant difference (*p* < 0.05).

The percentage of free ketone compounds in the headspace within the presence of MPs was determined as shown in Figure 6. The free flavour compounds in the vial without protein comprised 100%. If the percentage of free flavour compounds falls below 100%, it suggests the protein possesses an adsorption capacity for the flavour compounds. MPs showed an adsorption effect on four ketones [61]. In this study, the MPs showed an adsorption effect on four ketones. Generally, the molecular structure of flavour compounds plays an important role in the interaction between proteins and flavour compounds [62]. For the homologous series in ketone linearity, the flavour adsorption capacity of MPs to the four ketone compounds was in the order of 2-octanone > 2-heptanone > 2-hexanone > 2-pentanone, which is similar to some previous works [63]. This may be attributed to the enhanced hydrophobic interactions between proteins and long-chain ketone molecules, resulting in a great extent of hydrophobic binding between the proteins and the ketones; thus, the adsorption effect was more pronounced [64].

Untreated MPs (control) had a higher percentage of free ketones in the headspace, which is related to steric hindrance in the ability of MPs to bind flavour compounds [17]. Except for 2-octanone, the binding capacity of MPs at 20 min of ultrasound treatment only was significantly higher than that of the untreated group. This may be due to the cavitation effects of ultrasound, which led to the unfolding of proteins, the exposure of more active sites, and the interaction of more volatiles with proteins [17]. Only the addition of L-lysine significantly increased the binding capacity of MPs for ketone compounds (*p* < 0.05). This indicated that the addition of L-lysine can increase the adsorption capacity of MPs for ketones. The adsorption capacity was further increased by combining L-lysine with ultrasound, and the maximum adsorption was reached at 20 min. The adsorption capacity of 2-pentanone was increased from 4.91% to 22.16%, that of 2-hexanone was increased from 10.30% to 24.67%, that of 2-heptanone was increased from 15.51% to 22.38%, and that of 2-octanone was increased from 24.25% to 31.67%. The adsorption capacity achieved by MPs at 20 min was better than that of MPs treated with L-lysine alone without ultrasonic treatment (0 min) and with ultrasound alone.

The results showed that the combined treatment of ultrasound and L-lysine greatly improved the flavour adsorption capacity of MPs. When the MPs were treated with ultrasound combined with L-lysine, some of the protein particles were disaggregated, leading to the exposure of their active binding sites (hydrophobic and hydrogen binding sites), which increased the interaction with flavour compounds [65]. The combination of the two also enhanced the electrostatic effect, resulting in better binding of the MPs to compounds (Figure 3). At the same time, the secondary structure of the protein unfolds and the α-helix is disrupted, leading to an increase in the hydrophobic content of the surface and thus increasing the number of hydrogen bonding sites on the protein (Table 3) [66]. As a result, hydrophobic sites that are normally buried in the molecule were exposed, which enhanced the binding of MP to the flavour compounds. In addition, ultrasound combined with L-lysine treatment of MPs resulted in the unfolding of the tertiary structure and further exposure of the active binding sites, which increased the adsorption capacity for flavour compounds (Figure 5).

## 4. Conclusions

This study investigated the potential of combining ultrasound treatment with L-lysine to enhance the solubility, thermal stability, and flavour compound binding ability of MPs in low-ion-strength media. The results demonstrated that a 20 min ultrasound treatment combined with L-lysine achieved optimal dispersion stability. This effect can be attributed to the unfolding and depolymerization of MPs, which exposed SH groups and aromatic amino acids and induced a conformational transition from α-helix to β-turn. Additionally, this combined treatment also enhanced the adsorption capacity of MPs for kenone compounds by facilitating the formation of hydrophobic, hydrogen, and electrostatic bonding sites. In conclusion, the synergistic use of ultrasound and L-lysine offered a promising approach for enhancing the functionality of meat-based products, resulting in improved flavours and an increased utilisation of meat proteins.

## Figures and Tables

**Table 1:** Effects of ultrasound combined with L-lysine on the pH value of myofibrillar protein.

Ultrasound Time (min)	pH	
	Ultrasound	Ultrasound + L-Lysine
0	6.86 ± 0.02 ^b^	9.35 ± 0.02 ^a^
5	6.87 ± 0.01 ^b^	9.35 ± 0.03 ^a^
10	6.88 ± 0.02 ^b^	9.36 ± 0.01 ^a^
15	6.87 ± 0.01 ^b^	9.38 ± 0.01 ^a^
20	6.88 ± 0.01 ^b^	9.36 ± 0.01 ^a^

^a,b^—the same indexes and different letters differ significantly (*p* < 0.05).

**Table 2:** Effects of ultrasound combined with L-lysine on the particle size of myofibrillar protein.

Group	Particle Size (μm)				
	Ultrasound Time (min)	D_50_	D_90_	D_43_	D_32_
ultrasound	0	38.39 ± 0.73 ^bc^	137.63 ± 2.75 ^d^	61.12 ± 2.07 ^de^	22.76 ± 0.47 ^b^
	5	20.96 ± 1.37 ^cd^	74.41 ± 3.22 ^e^	33.94 ± 2.13 ^ef^	16.59 ± 1.12 ^bcd^
	10	15.63 ± 1.25 ^cd^	66.70 ± 0.72 ^e^	29.11 ± 1.06 ^f^	13.29 ± 1.16 ^bc^
	15	12.03 ± 3.92 ^d^	48.30 ± 2.98 ^e^	22.99 ± 4.08 ^f^	10.30 ± 2.69 ^d^
	20	11.03 ± 4.01 ^d^	28.80 ± 14.46 ^e^	15.89 ± 7.30 ^f^	9.79 ± 3.19 ^d^
ultrasound + L-lysine	0	86.67 ± 2.75 ^a^	528.48 ± 10.14 ^a^	184.04 ± 4.57 ^a^	39.63 ± 1.22 ^a^
	5	57.50 ± 13.82 ^b^	466.14 ± 28.58 ^b^	141.84 ± 8.21 ^b^	17.61 ± 3.75 ^bcd^
	10	57.04 ± 21.73 ^b^	200.95 ± 16.07 ^c^	90.59 ± 11.69 ^c^	19.65 ± 6.26 ^bc^
	15	56.60 ± 13.66 ^b^	167.65 ± 34.76 ^cd^	82.22 ± 16.99 ^cd^	19.61 ± 5.71 ^bc^
	20	38.99 ± 4.95 ^bc^	163.63 ± 12.47 ^cd^	75.16 ± 6.69 ^cd^	19.71 ± 1.31 ^bc^

D_90_: Particles smaller than it account for 90% of the particles. D_50_: The particle size that 50% of the sample particles are smaller than and 50% are larger than. D_32_: Surface-weighted mean particle size. D_43_: Volume-weighted mean particle size. ^a–f^—the same indexes and different letters differ significantly (*p* < 0.05).

**Table 3:** Effects of ultrasound combined with L-lysine on the secondary structures of myofibrillar proteins.

Group	Relative Content of Secondary Structures (%)				
	Ultrasound Time (min)	β-Sheet	Random Coil	α-Helix	β-Turn
ultrasound	0	20.58 ± 0.52 ^c^	22.65 ± 0.91 ^a^	24.49 ± 0.25 ^a^	32.28 ± 0.21 ^b^
	5	20.67 ± 0.23 ^bc^	22. 15 ± 0.17 ^a^	24.09 ± 0.37 ^ab^	33.09 ± 0.25 ^b^
	10	21.71 ± 0.59 ^bc^	21.98 ± 0.25 ^a^	23.74 ± 0.64 ^ab^	32.58 ± 0.36 ^b^
	15	22.70 ± 0.87 ^b^	21.41 ± 0.56 ^a^	23.49 ± 0.58 ^ab^	32.40 ± 0.51 ^b^
	20	28.57 ± 0.32 ^a^	22.39 ± 0.52 ^a^	22.18 ± 0.28 ^bc^	26.86 ± 0.56 ^c^
ultrasound + L-lysine	0	16.62 ± 0.29 ^d^	18.33 ± 0.32 ^b^	21. 10 ± 0.23 ^c^	43.94 ± 0.45 ^a^
	5	17.22 ± 0.43 ^d^	18. 16 ± 0.28 ^b^	20.79 ± 0.49 ^c^	43.83 ± 0.39 ^a^
	10	17.40 ± 0.52 ^d^	18.29 ± 0.24 ^b^	20.78 ± 0.36 ^c^	43.53 ± 0.27 ^a^
	15	17.41 ± 0.65 ^d^	18.70 ± 0.31 ^b^	21.08 ± 0.32 ^c^	42.80 ± 0.36 ^a^
	20	17.57 ± 0.53 ^d^	18.99 ± 0.56 ^b^	20.70 ± 0.21 ^c^	42.43 ± 0.62 ^a^

^a–d^—the same indexes and different letters differ significantly (*p* < 0.05).

**Table 4:** Effects of thermal denaturation temperature of pork myofibrillar proteins treated with ultrasound combined with L-lysine.

Ultrasound Time (min)	Denaturation Temperature (T_d_) (°C)	
	Ultrasound	Ultrasound + L-Lysine
0	98.6 ± 1.62 ^f^	109.5 ± 0.16 ^c^
5	100.3 ± 0.16 ^f^	109.9 ± 0.23 ^c^
10	104 ± 0.81 ^e^	112.5 ± 0.24 ^b^
15	107.3 ± 0.17 ^d^	113.6 ± 0.26 ^a^
20	112.1 ± 0.08 ^b^	114.5 ± 0.43 ^a^

^a–f^—in the same indexes and different letters differ significantly (*p* < 0.05).

## Data Availability

The data presented in this study are available on request from the corresponding author.

## Supplementary Materials

The following supporting information can be downloaded at: https://www.mdpi.com/article/10.3390/foods13040629/s1, Figure S1: Effect of L-lysine alone on the solubility of myofibrillar proteins. The letters above the bars indicate a significant difference (*p* < 0.05); Table S1: The probability level (*p*-value) for experimental main factor (ultrasound time and L-lysine) and their interaction effects on MP.
