# Supplementary material for: Synergistic Effects of High-Intensity Ultrasound Combined with L-Lysine for the Treatment of Porcine Myofibrillar Protein Regarding Solubility and Flavour Adsorption Capacity
Source: Foods. 2024 Feb 19;13(4):629. doi: 10.3390/foods13040629 (PMC10887734; doi:10.3390/foods13040629)
Supplement: Supplementary file 1 [file foods-13-00629-s001.zip › foods-2837063-supplementary.pdf]

Supplementary Material

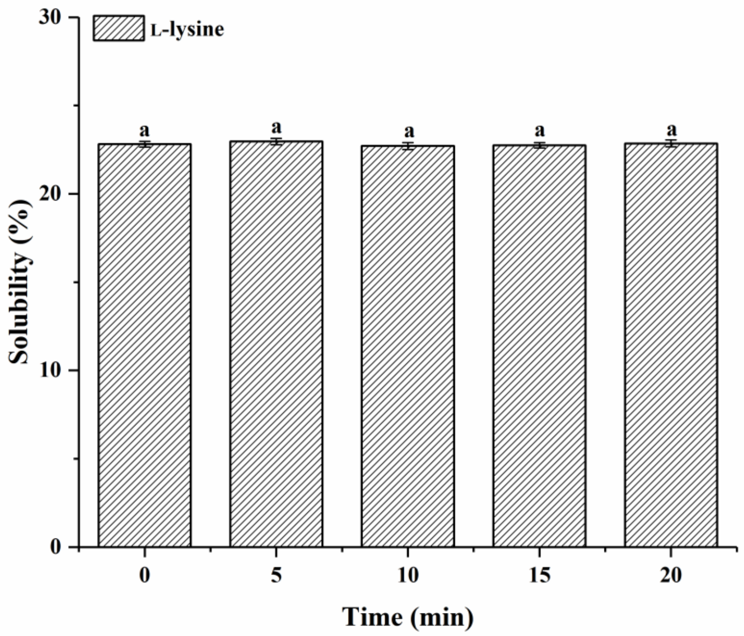

Figure S1. Effect of L-lysine alone on the solubility of myofibrillar proteins. The letters above the bars indicate a significant difference ($p < 0.05$).

Table S1. The probability level (p-value) for experimental main factor (ultrasound time and L-lysine) and their interaction effects on MP.

Experimental factor and interaction of experimental factors	Solubility	turbidity	Particle size of D_{43}	Reactive sulfhydryl	2-pentanone	2-hexanone	2-heptanone	2-octanone
ultrasound time	<0.05	<0.05	<0.05	<0.05	>0.05	>0.05	>0.05	>0.05
L-lysine	<0.05	<0.05	<0.05	<0.05	<0.05	<0.05	<0.05	<0.05
ultrasound time ×L-lysine	<0.05	<0.05	<0.05	<0.05	>0.05	>0.05	>0.05	>0.05
